# Crystal Violet Selectively Detects Aβ Oligomers but Not Fibrils In Vitro and in Alzheimer’s Disease Brain Tissue

**DOI:** 10.3390/biom14060615

**Published:** 2024-05-23

**Authors:** Kanchana Karunarathne, Teresa R. Kee, Hanna Jeon, Sara Cazzaro, Yasith I. Gamage, Jianjun Pan, Jung-A. A. Woo, David E. Kang, Martin Muschol

**Affiliations:** 1 Department of Physics, University of South Florida, Tampa, FL 33620, USA; 2 Department of Molecular Medicine, USF Health, Morsani College of Medicine, Tampa, FL 33620, USA; 3 Department of Pathology, School of Medicine, Case Western Reserve University, Cleveland, OH 44106, USA

**Keywords:** amyloid oligomers, amyloid beta, fluorescence, tissue staining, Alzheimer’s Disease, Thioflavin T, Crystal Violet

## Abstract

Deposition of extracellular Amyloid Beta (Aβ) and intracellular tau fibrils in post-mortem brains remains the only way to conclusively confirm cases of Alzheimer’s Disease (AD). Substantial evidence, though, implicates small globular oligomers instead of fibrils as relevant biomarkers of, and critical contributors to, the clinical symptoms of AD. Efforts to verify and utilize amyloid oligomers as AD biomarkers in vivo have been limited by the near-exclusive dependence on conformation-selective antibodies for oligomer detection. While antibodies have yielded critical evidence for the role of both Aβ and tau oligomers in AD, they are not suitable for imaging amyloid oligomers in vivo. Therefore, it would be desirable to identify a set of oligomer-selective small molecules for subsequent development into Positron Emission Tomography (PET) probes. Using a kinetics-based screening assay, we confirm that the triarylmethane dye Crystal Violet (CV) is oligomer-selective for Aβ42 oligomers (AβOs) grown under near-physiological solution conditions in vitro. In postmortem brains of an AD mouse model and human AD patients, we demonstrate that A11 antibody-positive oligomers but not Thioflavin S (ThioS)-positive fibrils colocalize with CV staining, confirming in vitro results. Therefore, our kinetic screen represents a robust approach for identifying new classes of small molecules as candidates for oligomer-selective dyes (OSDs). Such OSDs, in turn, provide promising starting points for the development of PET probes for pre-mortem imaging of oligomer deposits in humans.

## 1. Introduction

Alzheimer’s Disease (AD) is estimated to affect more than six million Americans, with as many as one in three seniors dying while suffering from AD [1]. Currently, there is no cure or effective treatment available. Despite intensive research efforts over multiple decades, there is still no universal agreement regarding the molecular mechanisms causing the clinical symptoms associated with AD. AD results in prominent deposits of extracellular Amyloid Beta (Aβ) fibril plaques and cytoplasmic neurofibrillary tau tangles in post-mortem brain biopsies [2]. Instead of these fibrillar deposits, though, one leading hypothesis for AD implicates small Aβ amyloid oligomers (AβOs) as the molecular toxin causing clinical symptoms [3,4,5,6,7,8]. AβOs induce cognitive deficits when injected in both rats and primates [9,10]. At the cellular level, AβOs alter long-term potentiation and long-term depression of synaptic transmission considered to be the cellular basis for memory formation and learning [11,12]. Furthermore, AβOs have been shown to disrupt an array of other cellular functions, including axonal transport, and to induce inflammation and oxidative stress [13,14,15,16]. In clinical trials, the recently FDA-approved antibody (Lecanemab), which preferentially targets Aβ oligomers and protofibrils over fibrils, has shown a modest but noticeable slowdown in disease progression, thereby further supporting the oligomer hypothesis [17,18].

The fibril indicator dye Thioflavin T (ThT), with its high sensitivity, ease of detection, and high temporal resolution for amyloid fibrils, highlights the benefits of fluorophores for amyloid detection [19]. Measurements of amyloid kinetics with ThT have been essential for developing detailed molecular models of amyloid fibril assembly [20,21,22]. Its uncharged variant Thioflavin S is a commonly used histological amyloid stain. Perhaps most significantly, ThT has served as a template for the amyloid binding moiety of Pittsburgh compound B, the first Positron Emission Tomography (PET) probe for imaging fibrillar plaques in vivo [23,24]. However, ThT binding is strongly skewed towards responses to late-stage fibrils over oligomers.

There are multiple biochemical and biophysical techniques available for detecting and characterizing AβOs formed in vitro, including atomic force microscopy [25,26,27], Photo-induced cross-linking of unmodified proteins (PICUP) [28], optical spectroscopy [29], mass spectroscopy [30], and, most prominently, immunostaining with conformation-dependent anti-oligomer antibodies [31,32]. Assessing the role of AβOs in the etiology of AD in vivo, however, has been hampered by two fundamental challenges. First, the build-up of Aβ deposits is estimated to precede the onset of clinical symptoms of AD by two decades or more [33]. At the same time, there are currently no reliable assays for measuring AβO populations in situ or for monitoring their spatial and temporal evolution. One potential solution would be the development of oligomer-selective probes for imaging AβO deposits in vivo using PET. Similar to the development of the currently available PET probes for fibril deposits, development of oligomer-selective PET probes would require the identification of small molecules with high selectivity for AβOs over monomers and fibrils. Due to the lack of detailed structural information on amyloid oligomers, a structure-based design approach for oligomer-selective small molecules is currently not feasible. Therefore, identification of selective binding to AβOs must rely on a rational and efficient screening protocol for promising candidates.

The standard approach for testing binding of potential oligomer-selective syes (OSDs) to immobilized monomers (AβM), oligomers (AβOs), or fibrils (AβFs) faces the difficulty of generating, isolating, and stabilizing the inherently transient and metastable AβOs for such an assay. Utilizing the kinetics of amyloid assembly, we have previously identified a different screening approach that circumvents the difficulties of isolating and immobilizing various amyloid aggregates. Specifically, we have noticed that in vitro amyloid growth of lysozyme and Aβ40 undergoes a prominent kinetic transition from sigmoidal fibril assembly with negligible oligomer populations to biphasic kinetics caused by lag-free formation of prominent oligomer populations [34,35,36]. Using this transition in lysozyme as a screening assay for oligomer-selective dyes, we have identified the triarylmethane dye Crystal Violet (CV) as a potential candidate [37]. However, the acidic pH and elevated temperature used during lysozyme amyloid growth in vitro have raised concerns about whether our approach would extend to AβOs formed by Aβ42. Here, we confirm that the same kinetic transition occurs during in vitro Aβ42 assembly and under near-physiological solution conditions. Second, we determine that CV remains similarly responsive to AβOs formed by Aβ42 (for simplicity referred to as AβOs from here on out) emerging under these growth conditions. Furthermore, we assess whether CV, as identified via this kinetic assay, continues to be selective in the presence of the multitude of other potential cellular binding targets in tissues. Here, we show that CV selectively stains AβOs in tissues from AD animal models, as well as from AD patients. This represents a critical step toward the development of CV into an oligomer-selective PET probe.

## 2. Materials and Methods

### 2.1. Aβ42 Preparation

Lyophilized Aβ42 peptide was acquired commercially (rPeptite, Watkinsville, GA or GL Biochem, Shanghai, China). Monomeric Aβ42, required as a starting point for the kinetic assay, was obtained by Fast Protein Liquid Chromatography (FPLC). One milligram of the lyophilized peptides was dissolved in 500 μL of 100 mM NaOH at pH 13 and injected into a Superdex 75 10/300 GL column (GE Healthcare, Chicago, IL, USA), equilibrated against a solution of 35 mM Na_2_HPO_4_ at pH 11. The monomer peak eluted near 14 mL after injection, was collected and immediately stored on ice. Concentrations of the resulting Aβ42 monomer stock were determined by integrating the UV absorption trace from the FPLC using a molar absorptivity of ε_280_ = 1470 M^−1^ cm^−1^ [38,39]. The typical yield was 1.5 mL of 40 µM to 60 µM Aβ42 monomers.

### 2.2. Dye Stock Preparation

Ultrapure grade Thioflavin T was purchased from Anaspec Inc. (Fremont, CA, USA). Crystal Violet was obtained from Thermo Scientific (Waltham, MA, USA). Dye stock solutions were prepared at a concentration of approx. 1 mM by dissolving the dye powder in 18 MΩ deionized water (Barnstead E-pure, Dubuque, IA, USA). Solutions were filtered consecutively through 220 nm and 50 nm syringe filters. Actual dye stock concentrations were determined from their optical absorption, using ε_412_ = 31,600 M^−1^ cm^−1^ for ThT [40] and ε_590_ = 87,000 M^−1^ cm^−1^ for CV [41], respectively.

### 2.3. Kinetic Screening Assay

A two-step procedure was used to prepare a series of Aβ42 solutions with concentrations typically ranging between 2 μM and 50 μM for kinetic growth experiments at pH 7.4 and 150 mM NaCl. Low-binding 1.5 mL microcentrifuge tubes were filled with 500 μL of 35 mM Na_2_HPO_4_ buffer and 150 mM NaCl at pH 11. The purified monomer stock was diluted into these tubes, with duplicates of solutions prepared for each Aβ42 concentration. A small amount (<5 μL) of 1 M HEPES buffer at pH 4.5 was added to adjust the final solution pH to 7.4. ThT (7.5 μL) or CV (5 μL) stock solutions were added to one of the two identical Aβ42 solution sets for a final dye concentration of 15 μM for ThT and 10 μM for CV. Three wells of a low-binding 96-well half-area microplate (#3881, Corning Life Sciences, Tewksbury, Massachusetts, MA, USA,) were filled with 150 μL of the various Aβ42/dye solutions. Plates were sealed with polyethylene sealing tape and incubated in a FLUOstar Omega fluorescence plate reader (BMG Labtech, Ortenberg, Germany) at 27 °C without shaking. Dye fluorescence was measured at 15 min. intervals for 1–2 days. For ThT fluorescence, a 448/10 nm excitation filter and a 482/10 nm emission filter were used. CV fluorescence was excited at 584/14 nm and measured using a 620/10 nm emission filter. Acquiring the fluorescence for either dye with both excitation/emission filter sets indicated that there was no overlap between ThT and CV emission. Dye/buffer wells without protein served as a control for potential changes in dye fluorescence (bleaching, hydrolysis, etc.) unrelated to protein aggregation.

### 2.4. AFM and TEM Imaging

A total of 50 μL of Aβ42 aliquots were deposited onto freshly cleaved mica substrate for 3–5 min. The excess solution was wicked off, rinsed with 1 mL of deionized water, and dried with a gentle stream of dry nitrogen. Amyloid aggregates were imaged in air with a Multimode-8 HR atomic-force microscope and SNL-10 tips with a nominal tip radius of 2 nm (both Bruker Corporation, Billerica, MA, USA). The cantilevers had a typical spring constant and resonance frequency of 0.24 N/m and 65 kHz, respectively. Images were acquired in quantitative nanomechanic mode (QNM) with a peak force of 300 pN at a scan rate of 0.5 Hz. Images were acquired at 256 × 256 pixels resolution. Height and phase images were collected for varying sample areas of 1 × 1 μm–5 × 5 μm. The image processing software Gwyddion v2.61 was used to collect the Z-height line profile for the images.

For TEM imaging, Aβ42 solutions were deposited onto Formvar/carbon film-coated 200 mesh copper grids (Electron Microscopy Sciences, Hatfield, PA, USA). Small drops (5 μL) were placed on the grid for 5 min., with the remaining solution wicked away with filter paper. The grid was negatively stained with 5 μL of 2% (*w*/*v*) uranyl acetate for 3–5 min. Excess uranyl acetate was removed by wicking it away with filter paper followed by repeated deposition/removal of distilled water. Grids were left to air dry for 5–10 min. before imaging with a FEI Morgagni transmission electron microscope at 60 kV connected to an Olympus MegaView III camera (Olympus, Tokyo, Japan).

### 2.5. Immunoblotting

Immunostaining with the oligomer-specific A11 antibody (generous gift from Prof. Rakez Kayed, Univ. Texas Medical Branch, Galveston, TX, USA) was used to verify the presence of oligomers/protofibrils in the solutions. All protein solutions were diluted to a uniform concentration of 2 μM prior to blotting. Diluted samples (2 μL) were blotted onto a nitrocellulose membrane (GE Amersham, Boston, MA, USA) prewetted with 1× TBS. The membrane was blocked with 7% milk in TBS for 1 h at room temperature, washed three times with 1× TBST for 5 min while gently agitated. Membranes were incubated overnight at 4 °C with the primary antibody A11 prepared in 7% milk in TBS with 0.02% sodium azide. The antibody was diluted 1:2000. The dot blot was washed and incubated for 1 h with a secondary Goat Anti-Rabbit IgG-HRP antibody (Southern Biotek, Birmingham, AL, USA) with 7% milk in TBS at 1:1000 dilution. Membranes were washed three times with 1× TBST and once with 1× TBS for 5 min each. Membranes were treated with ECL substrate chemiluminescence (Pierce Biotechnology Inc., Rockford, IL, USA) and imaged using a ChemiDoc XRS+ Gel Imaging System (Bio-Rad, Hercules CA, USA).

### 2.6. Mice and Tissue Processing

Mutant mice with a chimeric mouse/human amyloid precursor protein (Mo/HuAPP695swe) and a mutant human presenilin 1 (PS1-dE9), both directed to CNS neurons (APP^swe^/PS1^dE9^) mice [42] were obtained from Jackson Laboratories (B6;C3-Tg(APPswe,PSEN1dE9)85Dbo/Mmjax, JAX: 034829). Genomic DNA isolated from tail snips was used for genotyping by PCR with the following primers: forward primer: 5′-CTGACCACTCGACCAGGTTCTGGGT-3′, and reverse primer: 5′-GTGGATAACCCCTCCCCCAGCCTAGAC-3′. APP^swe^/PS1^dE9^ mice were bred and maintained in the C57BL/6J background. Water and food were supplied ad libitum with a 12 h light/dark cycle under standard vivarium conditions. All procedures involving mice were submitted to and approved by the Case Western Reserve University Institutional Animal Care and Use Committee (IACUC). Mice were perfused with 1× PBS and half brains were post-fixed with 4% paraformaldehyde (PFA) (Acros Organics, Geel, Belgium, 41678-5000) at 4 °C for 72 h followed by cryoprotection in 30% sucrose. After tissue saturation, brains were sliced in 25 μm sections and then processed for staining as previously described [43,44].

### 2.7. Mouse and Human Brain Staining for Aβ and A11-Positive Oligomers

Tissue staining was performed as previously described [43,44]. Briefly, tissues were washed three times for 10 min in 0.2% triton in 1× TBS. Epitope retrieval was performed in 0.5 M citrate buffer pH 6 (ThermoFisher, Waltham, MA, J63950) at 100 °C for 5 min, followed by two washes in 1× PBS. Tissues were then blocked for 1 h at room temperature in 3% goat serum and 0.2% triton X-100 in 1× PBS, followed by overnight incubation with anti-amyloid β (Cell Signaling Technology, Danvers, MA, USA, D54D2) or anti-oligomer A11 (from Rakez Kayed, UTMB) antibodies at a 1:500 dilution in blocking solution. The following day, tissues were washed three times in 1× PBS for 10 min and then incubated with Alexa Fluor secondary antibodies at 1:1000 dilution in blocking buffer for 45 min at room temperature. Following extensive washes in 1× PBS, sections were stained with CV.

### 2.8. Crystal Violet and Thioflavin S Staining

For CV staining, 1 mM CV stock in 20% MeOH in water was diluted to 1 μM in distilled water (1:1000 dilution). Tissues were then incubated in 1 μM CV for 10 min at room temperature. Following CV staining, tissues were washed five times with 1× PBS. If Thioflavin S staining was also performed, sections were washed in 70% EtOH for 1 min, followed by another 1 min wash in 80% EtOH. 1% Thioflavin S was freshly prepared prior to each use in 80% EtOH and filtered with a 0.2 μm filter. Sections were then incubated with Thioflavin S for 15 min, washed for 1 min in 80% EtOH, then 1 min in 70% EtOH, followed by two 1 min washes in distilled water. Slides were mounted and imaged with the Keyence BZ-X800 microscope with 391 nm/428 nm excitation/emission filters (for ThioS) and/or 590 nm/618 nm excitation/emission filters (for CV).

### 2.9. Data Analysis

Dye traces were exported to Igor data analysis software (Igor 8.0, Wavemetrics, Lake Oswego, OR, USA) for further analysis. By dividing sample fluorescence values F(t) by their corresponding dye/buffer fluorescence at the initial measurement time F(B0), the raw fluorescence data were converted into fractional fluorescence enhancements [F(t)/F(B0)]. Due to the absence of any significant drift in dye/buffer fluorescence, we did not perform a point-by-point buffer correction. For quantification of colocalization in APP/PS1 mouse and AD patient brains, the Fiji software (Java 6, NIH ImageJ, Bethesda, MD, USA) was utilized. Multichannel images were split into individual channels, the brightness/contrast was adjusted per channel to remove non-specific signals (as determined by negative controls), and the colocalization threshold program was used to calculate the Mander’s coefficient using thresholds (tM1/tM2) for different channel combinations (e.g., CV/A11, CV/ThioS). Statistical and graphical analyses were performed using GraphPad Prism software (version 8.0, La Jolla, CA, USA).

## 3. Results

### 3.1. Distinct ThT vs. CV Kinetics of Aβ42 Amyloid Growth Imply CV Is an Oligomer-Selective Dye

**Figure 1:**
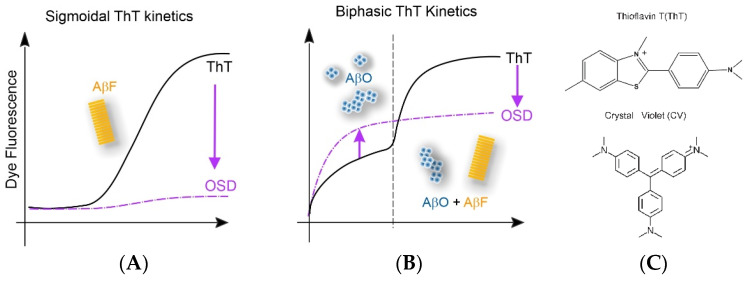
Schematic of kinetic dye assay to identify potential oligomer-selective dyes (**A**,**B**). Schematic of the transition in ThT kinetics from (**A**) fibril-dominated sigmoidal kinetics with a well-defined lag phase to (**B**) biphasic ThT kinetics with a lag-free, oligomer-dominated phase. The purple curve indicates that an Oligomer-Selective Dye (OSD) should display (**A**) a weak response during fibril-dominated sigmoidal growth but (**B**) a robust response to the oligomer-dominated phase of biphasic growth with only a weak or no response to the fibril-related secondary upswing. (**C**) Chemical structures of ThT and CV dye.

We have previously described a transition in ThT-monitored kinetics during in vitro amyloid assembly of Aβ40 and lysozyme [34,35] indicating the emergence of large populations of long-lived oligomers [34,35,36,37]. The ubiquitously observed sigmoidal ThT kinetics provide no signature of oligomer formation. In contrast, the onset of biphasic kinetics indicates the lag-free formation of significant oligomer populations superimposed on the sigmoidal ThT kinetics of fibril nucleation and growth. The emergence of a readily detectable oligomer signal in ThT during the lag phase of fibril growth, therefore, opens a distinct time window for observing oligomer formation in the absence of fibril nucleation and growth. Correspondingly, dyes that respond only to the initial, oligomer-dominated phase of biphasic growth but lack noticeable responses to either pure sigmoidal growth or the fibril-associated secondary upswing of biphasic kinetics are promising candidates for oligomers-selective dyes (see schematic in Figure 1). We used this transition in lysozyme to screen a small selection of known amyloid dyes, molecular rotors, and hydrophobic dyes for preferential responses to the oligomer-specific initial phase of biphasic kinetics. This identified the triarylmethane dye Crystal Violet (Figure 1C) as a potential oligomer-selective dye (OSD) [37]. Here, we investigated whether Aβ42 displays the same oligomer-induced transition from sigmoidal to biphasic ThT kinetics. In addition, we determined whether CV maintains its oligomer selectivity despite the distinct protein involved, the dramatically different solution conditions used for screening, and the pH-induced changes in CV charge state which underlie CV’s use as a colorimetric pH indicator.

**Figure 2:**
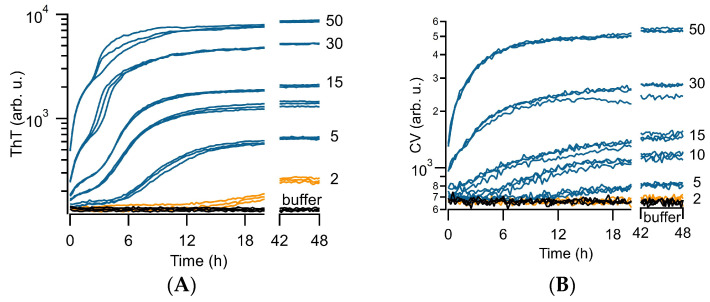

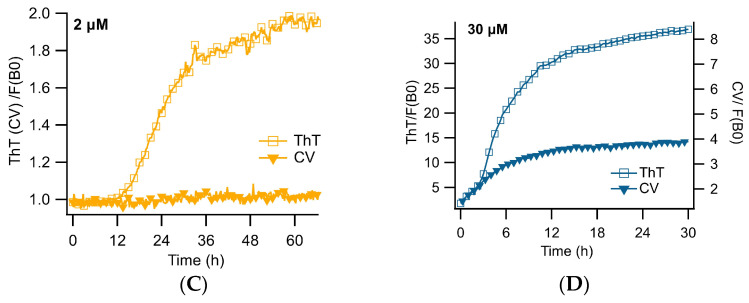
Thioflavin T vs. Crystal Violet kinetics during Aβ42 aggregation at pH 7.4. (**A**) Thioflavin T (ThT) vs. (**B**) Crystal Violet (CV) fluorescence responses during growth of Aβ42 at the indicated concentrations (in μM) at pH 7.4, 150 mM NaCl at 27 °C. Shown are three dye traces collected for each protein concentration. Thioflavin T displays the transition from fibril-dominated sigmoidal kinetics (orange traces) at 2 μM to progressively more oligomer-induced biphasic kinetics (blue traces), highlighted by the use of semi-log plots. Crystal Violet responds to the oligomer-dominated phase of biphasic ThT kinetics but lacks responses to the delayed secondary upswing due to accelerating fibril kinetics. (**C**,**D**) Overlay of the fractional changes in ThT vs. CV fluorescence during (**C**) fibril-dominated sigmoidal growth at 2 μM and (**D**) prominent biphasic growth at 30 μM. CV shows no discernible response to fibril growth (2 μM) but perfectly tracks the initial oligomer phase of biphasic ThT kinetics, which persists deep into the fibril-dominated secondary phase. We have previously shown that biphasic ThT kinetics is due to the superposition of lag-free and rapidly increasing formation of off-pathway oligomers with sigmoidal fibril kinetics [35,36] The persistent CV responses deep into the fibril growth phase match with the continued presence of AβOs observed with TEM and oligomer-selective antibodies (see Figure 3).

As shown in Figure 2A, in vitro growth of Aβ42 in HEPES buffer at pH 7.4 and 150 mM NaCl at 27 °C replicated the transition in ThT kinetics. Below the transition, the ubiquitous sigmoidal ThT kinetics of amyloid assembly is observed: an extended lag phase is followed by a sudden upswing in ThT before reaching a new plateau value (Figure 2C). Such sigmoidal kinetics are well-reproduced by models of nucleated polymerization [45,46]. As Aβ42 concentrations increased beyond a certain threshold, ThT-monitored kinetics consistently switched from purely sigmoidal to progressively biphasic kinetics. The lag-free primary phase of biphasic growth was highly reproducible, while the secondary upswing retained some of the statistical variability expected for nucleation-limited fibril growth (Figure 2A). As previously shown, and confirmed in the next section, this primary phase of biphasic growth is associated with a steep increase in off-pathway oligomer formation with protein concentration. In contrast to biphasic kinetics observed with Aβ40 [34,35], Aβ42 showed considerably shorter delays for the onset of the secondary, fibril-related phase of biphasic kinetics (Figure 2A). Figure 2B displays the corresponding fluorescence kinetics of Aβ42 obtained with CV under identical conditions. In comparison to ThT, CV only yielded a noticeable response to the primary phase of biphasic kinetics but lacked the upswing of the secondary phase by ThT. Equally important, CV responses did not diminish but persisted deep into the fibril nucleation and growth phase. An additional four sets of ThT vs. CV responses during the growth of Aβ42 under these conditions are shown in Supplemental Figure S1.

To further analyze the correlation between ThT and CV responses during sigmoidal vs. biphasic growth, we superimposed the fractional changes in ThT vs. CV kinetics (Figure 2C,D). At Aβ42 concentrations below the kinetic transition (~2–5 μM), ThT displayed the expected sigmoidal kinetics associated with nucleated polymerization of fibrils. In contrast, CV showed no discernible response over the entire growth period (Figure 2C). It is worth noting that neither ThT nor CV could detect the small fraction of short-lived on-pathway oligomers and primary nucleation events that must occur during the lag phase. During biphasic growth ([Aβ42] ~> 5 μM), the primary phase of ThT kinetics could be perfectly matched to the same segment of CV kinetics, but rapidly diverged upon the onset of the secondary phase (Figure 2D). All of the above features align with our prior observations of ThT and CV during lysozyme amyloid growth at pH 2 [37].

### 3.2. CV Kinetics Match with the Presence of Aβ Oligomers in Solution

In the above discussion of ThT vs. CV fluorescence kinetics, we had implicitly assumed that the kinetic transition of ThT from sigmoidal to biphasic kinetics in Aβ42 would replicate the non-linear and lag-free increase from off-pathway oligomer and protofibril formation we had previously described for Aβ40 and lysozyme [34,35,36]. To confirm that ThT and CV kinetics correlate with the formation of biphasic AβOs in Aβ42, we used two approaches: high-resolution Atomic Force Microscopy (AFM) and Transmission Electron Microscopy (TEM) imaging of aggregate morphologies and time-resolved and concentration-dependent staining of samples with the oligomer-selective antibody A11 [32,47].

**Figure 3:**
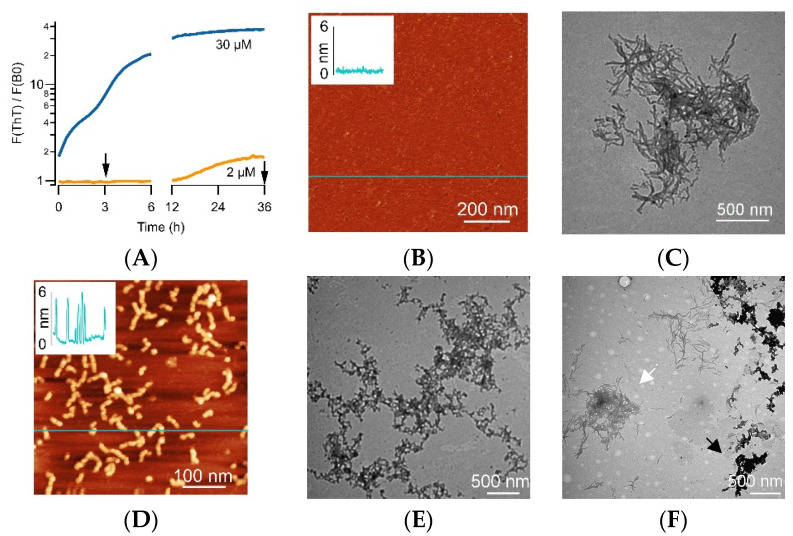
Aggregate morphologies in the sigmoidal vs. biphasic pathway. (**A**) Representative sigmoidal (orange, 2 μM) and biphasic (blue, 30 μM) ThT kinetics of Aβ42, with arrows indicating typical early stage (~3 h) and endstage (36+ h) time points for aliquots collected for AFM and/or TEM imaging. (**B**,**C**) Sigmoidal pathway (2 μM): AFM images during the lag phase (**B**;~3 h) yielded no discernible protein aggregates (inset: typical height profile at line indicated in image). End-stage TEM images (**C**) displayed mixtures of isolated and bundled fibrils. (**D**–**F**) Biphasic pathway (5–50 μM): AFM (**D**) and TEM (**E**) images of oligomeric/protofibrillar aggregates observed during the initial phase (≤3 h) of biphasic kinetics (inset: height profile at the scan line indicated in the AFM image). The images show the correspondence of the beads-on-a-string morphology for oligomers/protofibrils in AFM and TEM imaging modalities. Late-stage TEM images (>36 h) (**F**) yielded mixtures of darkly stained oligomeric/protofibrillar aggregates (dark arrow) together with lightly stained and increasingly clumped fibrils (white arrow).

The arrows in Figure 3A indicate the typical time points for collecting aliquots for imaging. During sigmoidal growth (2 μM), AFM images of aliquots taken near the 3 h mark yielded no discernable aggregates (Figure 3B). The TEM images of the corresponding late stages (>36 h) showed individual fibrils or fibril bundles (Figure 3C). In contrast, AFM images from early-stage solutions displaying biphasic kinetics (5–50 μM) contained either individual oligomers or oligomers merged into “beaded” protofibrils (Figure 3D). TEM images taken at the same time point showed darkly stained protofibrils (Figure 3E). TEM images of the corresponding late stages showed mixtures of darkly stained protofibrils and lightly stained fibrils and fibril bundles (Figure 3F). The presence of protofibrils at the late stages correlates with the persistence of the CV fluorescence deep into the fibril growth and nucleation regime and underscores the metastability of these off-pathway oligomer populations.

**Figure 4:**
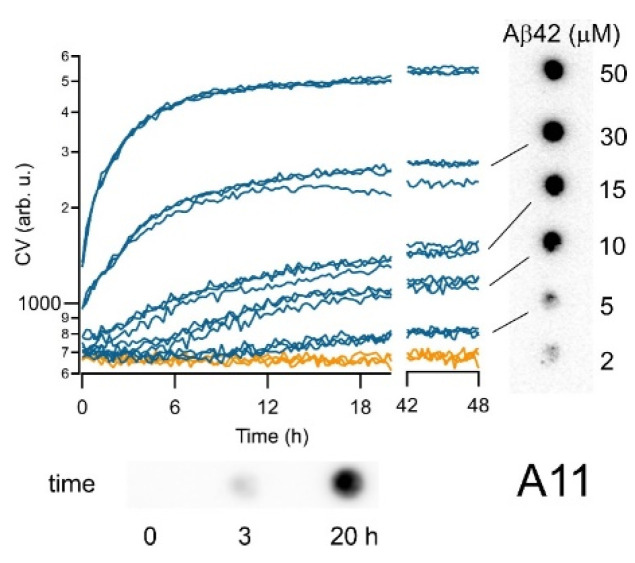
CV kinetics correlates with immunostaining against Aβ42 oligomers. The persistence and amplitude of the CV fluorescence (orange: no oligomers; blue: oligomer response; data from Figure 2B) correlates with immunostaining with oligomer-selective antibody A11 at the end of the incubation period (right side). Note that Aβ42 aliquots were all diluted to 2 μM concentration prior to blotting. In addition, aliquots from a 60 μM sample, sampled at 0 h, 3 h, and 20 h and stained with A11 (bottom panel), match with the build-up of oligomer populations well past the lag phase observed with ThT under those conditions (Figure 2A).

To correlate CV kinetics (Figure 4, main panel) with AβO populations, we stained all end stages of the samples with the oligomer-selective antibody A11 (generous gift from Dr. Rakez Kayed). To account for the differences in Aβ42 concentrations, we first diluted all solutions to the lowest concentration in our experiments (2 μM) before blotting them onto the membranes. As seen in Figure 4 (right panel), A11 showed next to no response at the lowest Aβ42 concentration but confirmed the presence of increasingly prominent populations of AβOs at the end stage of all biphasic growth conditions. The bottom in Figure 4 documents the evolution of A11 staining at the beginning (0 h), within the primary phase (3 h), and deep into the secondary phase (20 h) during biphasic Aβ42 growth (60 μM). The temporal increase in A11 responses matched well with the increasing CV fluorescence throughout the biphasic growth (Figure 4, main panel). Therefore, CV kinetics, TEM images, and immunostaining with A11 all indicate the formation of oligomers during the primary phase of biphasic kinetics, as well as the persistence of biphasic AβOs deep into the fibril-related secondary phase of biphasic ThT kinetics. Furthermore, the correlation between the persistent CV fluorescence deep into the fibril growth regime of Aβ42 with the presence of AβOs/protofibrils seen in TEM images and with A11 immunoblotting implies that CV faithfully monitors AβO kinetics independent of fibril growth. Overall, our results imply that CV has noticeable selectivity for A11 responsive, biphasic Aβ42 oligomer over their corresponding fibril populations.

### 3.3. CV Selectively Detects AβOs over AβFs in APP/PS1 Mouse and Human AD Brains

**Figure 5:**
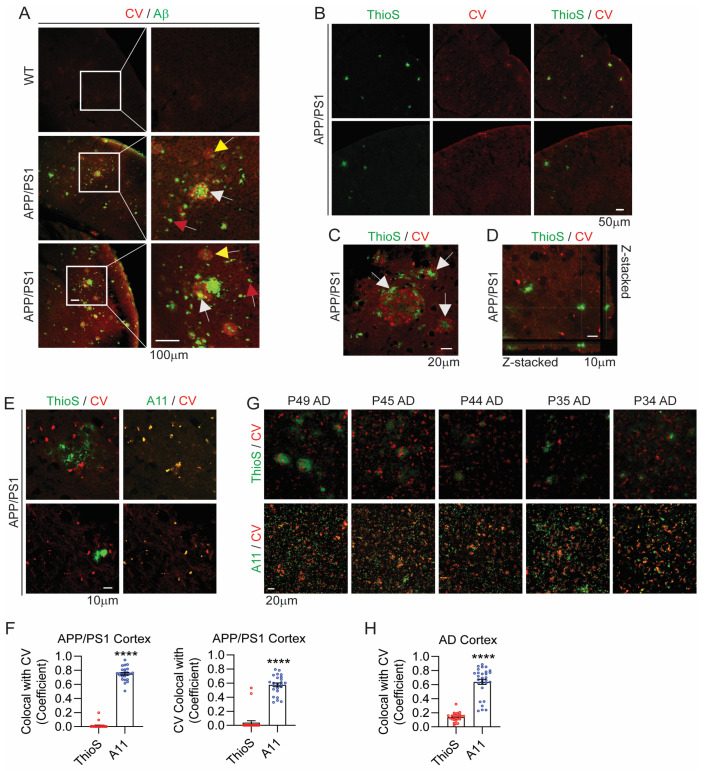
CV selectively detects AβOs over AβFs in APP/PS1 mouse and human AD brains. (**A**) Representative images of 8-month-old APP/PS1 and WT littermate mouse brains stained with Aβ antibody (D54D2, Cell Signaling) (green) and CV (red). White boxes magnified to the right. White arrows: examples of Aβ antibody-stained plaques with major CV staining; red arrows: examples of Aβ antibody-stained plaques with little CV staining; yellow arrows: examples of CV-stained plaques with little Aβ antibody immunoreactivity. (**B**) Representative images of 8-month-old APP/PS1 mouse brains stained with Thioflavin S (ThioS, green) and CV (red). (**C**) A representative image of an APP/PS1 mouse brain section showing clear juxtaposition of CV staining to ThioS+ fibrils but without colocalization (white arrows). (**D**) Z-stacked image of a ThioS+ plaque lacking CV staining. (**E**) Representative images of 8-month-old APP/PS1 mouse brain sections stained with oligomer-selective A11 antibody, CV, and ThioS. (**F**) Quantification of ThioS and A11 colocalization with CV (left graph) and CV colocalization with ThioS and A11 (right graph) (*t*-test, **** *p* < 0.0001, n = 25 cortical images/group from 2 APP/PS1 mice). (**G**) Representative images of ThioS/CV and A11/CV staining from the frontal cortex of five AD patient brains. (**H**) Quantification of ThioS and A11 colocalization with CV in AD brains (*t*-test, **** *p* < 0.0001, n = 26–29 images/group from five AD patient brains).

While the above results establish that CV is structurally selective for biphasic AβOs, we next determined whether it detects Aβ deposits and maintains structural selectivity for AβOs over AβFs in mouse and human brain tissues. To determine whether CV detects Aβ deposits, we stained the brains of 8-month-old APP/PS1 [48,49] and wild-type (WT) littermate mice for Aβ (D54D2 antibody, Cell Signaling) followed by CV staining. Indeed, abundant Aβ deposits (green) were detected in APP/PS1 mice, but such deposits were absent in WT mice (Figure 5A, top panel). Likewise, CV staining (red) in WT mice showed an indistinct diffuse staining pattern (Figure 5A, top panel). By contrast, CV staining (red) was more intense in APP/PS1 brains and generally colocalized with Aβ deposits (green) (Figure 5A, white arrows). However, some Aβ immunoreactivity contained little to no CV staining (Figure 5, red arrows), indicating that CV does not detect all forms of Aβ. Further, CV also detected some plaques that were weakly immunoreactive for Aβ, suggesting that the D54D2 Aβ antibody also does not detect some forms of Aβ (Figure 5A, yellow arrows).

Next, we stained 8-month-old APP/PS1 mouse brains with CV followed by Thioflavin S (ThioS), the latter detecting fibrillar amyloid. ThioS positive (ThioS+) fibrils (green) were nearly completely devoid of CV staining (red) (Figure 5B–F). Closer examination of plaque-like deposits showed intense ThioS+ fibrils often in plaque cores that were juxtaposed to CV staining but not colocalized with it (Figure 5C, white arrows). Z-stacked images confirmed that ThioS+ plaques did not colocalize with CV staining (Figure 5D, Supplemental Video S1). By contrast, triple staining with the oligomer-selective A11 antibody (green), CV, and ThioS showed that A11 staining (green pseudo-colored) nearly completely colocalized with CV staining (red) (Figure 5E), confirming that CV labels oligomers but not fibrils. The negative control using the secondary antibody only did not show any detectable immunoreactivity (Supplemental Figure S2). Quantification by Image J indeed showed colocalization coefficients of ~0.75 for A11 colocalization with CV and ~0.57 for CV colocalization with A11, whereas colocalization coefficients were ~0.014 and ~0.039, respectively, for ThioS (Figure 5F), demonstrating remarkable selectivity of CV to detect oligomers vs. fibrils. Similarly, CV and ThioS staining of the frontal cortex of AD brains showed little colocalization, whereas A11 staining colocalized remarkably well with CV staining with a coefficient of ~0.64 (Figure 5G,H; Supplemental Table S1, AD case info). These results confirm the selectivity of CV for AβOs over AβFs not only in an amyloid model of AD, but also in clinically relevant human AD brain tissue.

## 4. Discussion

Our results indicate that the transition from fibril-dominated sigmoidal to oligomer-induced biphasic ThT kinetics, previously described for lysozyme and Aβ40, extends to Aβ42 [35,36]. Consequently, the kinetic screening assay for OSDs we have reported earlier can be extended to Aβ42 as well [37]. We further provide evidence that the previously identified dye CV displays selectivity for AβOs over AβMs and AβFs of Aβ42. More importantly, CV exhibits significant selectivity for clinically relevant AβO populations over AβMs, AβFs, and other tissue components in mouse models of AD as well as tissues from AD patients. Hence, CV and A11 recognize biphasic AβOs grown in vitro and AβOs present in tissues. Given the large difference in solution conditions, the changes in the charge of CV with pH, and the lack of any structural similarities between lysozyme and Aβ42 monomers, it is perhaps surprising to see that CV retains its selectivity for AβOs of Aβ42. This suggests that CV, similarly to ThT for fibrils, binds to a generic structural feature of amyloid oligomers. We therefore anticipate that CV will recognize amyloid oligomers formed by a wide variety of amyloid proteins such as tau, aSyn, huntingtin, or amylin. It is intriguing to note that CV has been described as an amyloid-specific dye for tissue staining but was traditionally considered an inferior alternative to ThioS [50,51]. This might well have been due to the muted CV response to amyloid fibrils, which are the most common histological features evaluated in amyloid disease histology.

There are immediate applications for oligomer-selective fluorophores for in vitro studies of amyloid aggregation. As highlighted in Figure 2D, CV allows for the monitoring of the evolution of oligomer populations with a temporal resolution not feasible with antibodies, and concurrently with fibril formation. CV should also simplify the detection and quantification of oligomer populations in high-resolution optical microscopy, such as total internal reflection microscopy [52] or superresolution microscopy [53]. This should help address the question of whether the large populations of amyloid oligomers readily observed in vitro are on-pathway precursors or off-pathway competitors of fibril formation [54].

Histological staining with CV significantly simplifies and speeds up the process of detecting oligomers in tissues when compared to the multi-step incubation required for immunostaining. The low cost, ready availability, purity, and simple chemical structure of CV also compare favorably to the cost of antibodies and variability in their quality. Another encouraging aspect of our findings is the uniformity and modest amount of background fluorescence of CV in WT mouse brains (Figure 5A, top row). This suggests that CV, comparably to ThT, preferentially binds to oligomers not just in vitro, but also compared to other tissue components. As a result, neuropathological staining should be able to assess not only fibrils but also oligomers, as well as their spatial and temporal evolution, in model animals as well as patient tissues. This is further simplified by CV’s wide separation of its excitation/emission peaks from those of ThT and ThioS. Therefore, it can be readily used simultaneously with ThioS, as done in this study.

Our long-term objective, however, is to identify OSDs for potential development into PET probes. Its small size and its oligomer selectivity described here make CV a promising candidate for evaluation as an oligomer-selective PET probe. The encouraging results of recent clinical trials of antibody treatment for AD with an oligomer/protofibril-selective antibody provide significant impetus for the development of such a PET probe for selective detection of AβO populations in vivo. There are several other dyes reported to have oligomer selectivity [55,56,57,58]. Of these, custom-designed derivatives of oligothiophene have been evaluated in vitro and in tissues with distinct specificity for fibrils vs. oligomers [59]. The potential of any of these OSDs as an oligomer-selective PET probe, however, will require evaluation of a multitude of additional features, including their biocompatibility and bio-accessibility, probe uptake and clearance, and background signals from non-specific binding.

## Data Availability

Original data available from the authors upon request.

## Supplementary Materials

The following supporting information can be downloaded at: https://www.mdpi.com/article/10.3390/biom14060615/s1, Video S1: ThioS+ staining does not co-localize with CV; Figure S1: additional ThT vs. CV kinetics. Figure S2: Negative control staining with secondary antibody; Table S1: AD case information.
