# Supplementary material for: Crystal Violet Selectively Detects Aβ Oligomers but Not Fibrils In Vitro and in Alzheimer’s Disease Brain Tissue
Source: Biomolecules. 2024 May 23;14(6):615. doi: 10.3390/biom14060615 (PMC11201545; doi:10.3390/biom14060615)
Supplement: Supplementary file 1 [file biomolecules-14-00615-s001.zip › biomolecules-2963107-supplementary.pdf]

Crystal Violet Selectively Detects Aβ Oligomers but not Fibrils,

In Vitro and in Alzheimer's Disease Brain Tissue

Figure S1: ThT vs CV Kinetics

Matched pairs of **A**) Thioflavin T (top) *vs*. (**B**) Crystal Violet (bottom) fluorescence kinetics during growth of Aβ42 at the indicated concentrations (in μM) at pH 7.4, 150 mM NaCl at 27°C. Data represent four independent repeats of the experiments in addition to data shown in Fig. 2. Each dye trace represents the average from three wells at the same protein concentration.

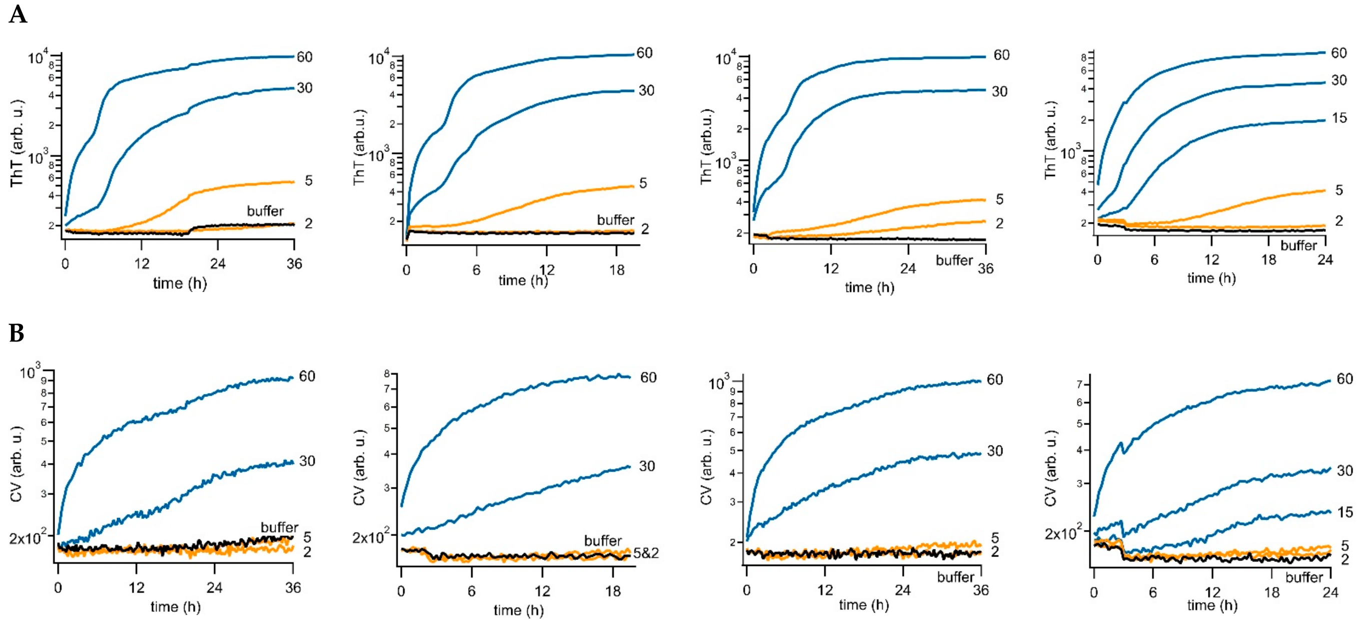

Figure S2. Negative control staining with secondary antibody

Negative control staining with secondary antibody (green) without primary antibody and CV (red), showed no staining with secondary antibody or co-staining with CV.

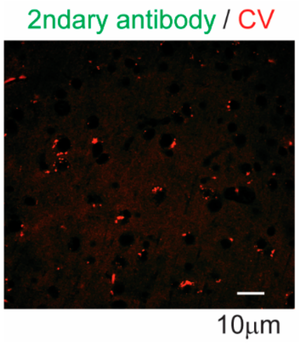

Video S1: ThioS+ staining does not co-localize with CV

Supplemental Video S1.avi

Video of Z-stacked images showing the juxtaposition, but lack of co-localization, of ThioS+ plaque vs. CV staining for APP/PS1 brain slice shown in Fig. 5D.

Table S1 AD case information

Floating sections of paraformaldehyde-fixed frontal gyrus cortex brain tissues from AD cases were obtained from the Alzheimer's Disease Research Center at Emory University. Available information on Braak stage, ABC Score, CERAD score, Thal score, APOE genotypes, ethnicity (w=White, b=Black, h=Hispanic), sex (m=male, f=female), post-mortem interval (PMI) [illegible]

Patient Number	Case Number	Primary Neuropathologic Diagnosis	Braak stage	ABC Score	CERAD Score	Thal Score	PMI (hr)	Age at Onset	Age at Death/Bx	Duration	ApoE	Race/Sex
34	OS00-25	AD	VI	High	Frequent	5	na	57	63	6	E3/4	w/m
35	OS00-32	AD	VI	High	Frequent	5	3.5	55	62	7	E3/4	w/m
44	E04-186	AD	VI	High	Frequent	5	7	59	72	13	E3/4	w/f
45	E05-04	AD	VI	High	Frequent	5	4.5	52	64	12	E3/4	w/f
49	E06-155	AD	VI	High	Frequent	5	6.5	56	67	11	E2/3	w/m
